# A purely bioinformatic pipeline for the prediction of mammalian odorant receptor gene enhancers

**DOI:** 10.1186/s12859-019-3012-1

**Published:** 2019-09-14

**Authors:** Andrea Degl’Innocenti, Gabriella Meloni, Barbara Mazzolai, Gianni Ciofani

**Affiliations:** 1 Smart Bio-Interfaces, Istituto Italiano di Tecnologia, Viale Rinaldo Piaggio 34, 56025 Pontedera (Pisa), Italy; 2 Max Planck Institute for Biophysics, Max-Planck-Gesellschaft, Max-von-Laue-Straße 3, D-60438 Frankfurt am Main, Germany; 3 Center for Micro-BioRobotics, Istituto Italiano di Tecnologia, Viale Rinaldo Piaggio 34, 56025 Pontedera (Pisa), Italy; 4 The BioRobotics Institute, Scuola Superiore Sant’Anna, Viale Rinaldo Piaggio 34, 56025 Pontedera (Pisa), Italy; 5 Department of Mechanical and Aerospace Engineering, Politecnico di Torino, Corso Duca degli Abruzzi 24, 10129 Torino, Italy

**Keywords:** Odorant receptor, Vomeronasal receptor, Odorant receptor gene choice, Enhancer, Element, Prediction, Cluster, Solitary gene, Minicluster, Sfaktiria

## Abstract

**Background:**

In most mammals, a vast array of genes coding for chemosensory receptors mediates olfaction. Odorant receptor (OR) genes generally constitute the largest multifamily (> 1100 intact members in the mouse). From the whole pool, each olfactory neuron expresses a single OR allele following poorly characterized mechanisms termed *OR gene choice*. OR genes are found in genomic aggregations known as *clusters*. Nearby enhancers, named *elements*, are crucial regulators of OR gene choice. Despite their importance, searching for new elements is burdensome. Other chemosensory receptor genes responsible for smell adhere to expression modalities resembling OR gene choice, and are arranged in genomic clusters — often with chromosomal linkage to OR genes. Still, no elements are known for them.

**Results:**

Here we present an inexpensive framework aimed at predicting elements. We redefine cluster identity by focusing on multiple receptor gene families at once, and exemplify thirty — not necessarily OR-exclusive — novel candidate enhancers.

**Conclusions:**

The pipeline we introduce could guide future in vivo work aimed at discovering/validating new elements. In addition, our study provides an updated and comprehensive classification of all genomic *loci* responsible for the transduction of olfactory signals in mammals.

**Electronic supplementary material:**

The online version of this article (10.1186/s12859-019-3012-1) contains supplementary material, which is available to authorized users.

## Background

Olfaction is the sense through which airborne or waterborne chemicals are detected and perceived as odors. The archetype of the mammalian olfactory system is located in the upper respiratory tract of the head, and possesses two principal sensory structures: the main olfactory epithelium (MOE), covering part of the nasal cavities and responsible for the detection of most odorants, and the vomeronasal organ (VNO), a hollow structure harboring an epithelium that specializes in sensing pheromones. Along with these two, minor sensory organs are the Grueneberg ganglion and the septal organ [1–3] (also reviewed in [4, 5]). Compared to mouse or rat, the human olfactory system presents a simplified organization that reflects a proportionally reduced importance of smell for survival, cf. [6]. The VNO, for instance, is only sometimes present in humans, and it is largely considered a vestigiality, e.g. [7].

Within the sensory epithelia of the olfactory system, sensory transduction relies on specialized neurons. Their dendrites — provided with sensory cilia — are embedded in a mucus that coats the airways, and through which environmental chemicals are trapped. Sensory neurons of the MOE are called olfactory sensory neurons (OSNs), while those of the VNO are termed vomeronasal sensory neurons (VSNs) [8–10] (cf. [4, 5]). OSNs and VSNs express sensory G protein-coupled receptors (GPCRs) that belong to extremely large subgroups, respectively odorant receptors (ORs) [11] and vomeronasal receptors (VRs) [12–15]. OR genes constitute the vastest mammalian gene multifamily, comprising in the mouse almost 1400 members, of which around 1100 intact [11, 16]. The human array of OR genes, though shrank and strongly pseudogenized, is still remarkable; it includes 800 genes, half of which are thought to be functional [16]. OR genes can be further divided in class I (fish-like, less abundant) and class II [17].

Central to the study of olfaction genetics is the so-called *one-neuron one-receptor* rule: through still elusive molecular mechanisms, often referred to as *OR gene choice*, each OSN expresses a single OR allele out of the whole repertoire, so that the expression domain of each OR gene in the MOE has a *punctate* (i.e. dotted) appearance. Namely, two adjacent OSNs would almost never pick up for expression the same OR gene [18–20]. As it is often the case for monoallelically or monogenically expressed gene families, OR genes are not uniformly dispersed in the genome. They are rather mostly arranged next to each other within a limited number of *loci* [21], allegedly with a mean intergenic distance of ~ 25 Kb [22, 23]. The mouse genome contains roughly fifty OR gene clusters [16], plus a few isolated OR genes (termed *solitary*) [17, 23–25].

The particular genomic arrangement of OR genes is likely a necessary condition to fulfill their expression requirements: sets of genes in the same *locus* share regulatory features. Notably, there are a number of gene enhancers — each found within or at least in proximity to an OR cluster — known to regulate OR gene expression *in cis* [24, 26–28], see also [29–32]. Prior to OR gene choice, OR chromatin is densely packed via repressive epigenetic markings; as the OSN matures, a single OR gene gets de-silenced and its expression is established [33, 34]. This probably occurs mainly thanks to a local fold in the DNA that causes an OR promoter to bind a flanking enhancer (reviewed in [35]). Both OR promoters and enhancers (better known as *elements* [26]) are AT-rich sequences containing, among other conserved motifs, transcription factor binding sites (TFBSs) for homeodomain (HD) and olfactory/early B (O/E) transcription factors. In addition, both entities possess each a typical epigenetic signature [28, 30, 32, 36, 37], see also [38]. Although specific OR genes can be selected by OSNs only within a certain expression domain of the MOE, randomness is still thought to play a major role in the process [21, 39, 40].

The first elements discovered, and still the most characterized, are H [26, 37] and P [27, 41]. A second, conspicuous group of enhancers was proposed with various degree of confidence by Markenscoff-Papadimitriou et al. [28]: of these, an element called Lipsi is robustly confirmed in vivo by knock-out mice; two more sequences, Kefallonia and Sfaktiria, are currently supported by reporter mouse strains. An additional element (named J) was recently found, to date the only one confirmedly regulating class I OR genes [42]. Elements have been almost exclusively studied in the mouse, and formally demonstrated invariably through mouse genetic engineering. Yet, many of them display some degree of cross-species conservation: H indeed stands for *homology*, because the sequence was initially identified by comparing human and mouse DNA [26].

OR and VR genes share many properties, e.g. VR genes are also clustered and expressed with oligogenic modalities. In the mouse, of the two subtypes of VR genes — V1R and V2R genes — V1R genes (around 300 genes, almost half of which pseudogenized) are expressed monoallelically and monogenically by apical VSNs [12, 43–47]. V2R genes are instead expressed by basal VSNs, according to a more complex paradigm: there are four subfamilies of V2Rs, known as A, B, D (together around 120 intact genes out of about 280) and C (seven members); each basal VSN chooses a single allele of subfamily C plus one or more V2R genes belonging to A, B or D [13–15, 48–51]. The human VR repertoire appears to be almost completely non-functional [47, 52, 53].

For some OSNs, trace amine-associated receptors (TAARs) can surrogate ORs. TAAR genes code for chemosensory GPCRs, and are arranged as a single cluster in a number of different mammalian species [54]. There are other, even less typical types of OSNs: they belong to a subsystem known as *necklace*; these sense pheromones by expressing multiple members of the so-called membrane spanning 4A gene family, coding for non-GPCR chemoreceptors [55]. As TAARs in the MOE, formyl peptide receptors (FPRs) are GPCRs that can replace VRs for some sensory neurons of the VNO, mainly apical VSNs. Their genes (seven in the mouse) are found within a broad but single genomic region, and display monogenic expression as well [56, 57].

The genomic *architecture*, i.e. organization, of OR and related genes appears to be deeply connected with their regulation. There are indeed few doubts that clusters constitute not only physical, but also functional units. Defining cluster identity and boundaries is therefore crucial when one wishes to discover *cis*-acting enhancers. We do know, for example, that OR and VR clusters tend to be located near to each other; while this might account for some of the similarities between OR and VR gene expression, at present not a single element is known to be shared between OR and VR genes, and in general no VR enhancers have been discovered. Also, among other known analogies between the olfactory and the immune system, a strong genomic linkage is present between OR and immune *loci*. T-cell receptor (TCR) gene segments, for instance, are systematically found in proximity to OR genes [58]. They also display oligogenic, stochastic expression, and are organized as clustered gene fragments, cf. [59]. The existence of shared regulatory sequences for the two gene families would at least be plausible, still none has been identified thus far.

Khan et al. [27] forecast ~ 150 elements for the mouse, and recent estimates rose the current number of candidate OR enhancers to about eighty [60]. Even more prudent predictions would concede that the hunt for elements is still in its dawn and infancy. While some of the last contributions undoubtedly represented a breakthrough in olfaction genomics, searching for elements remains an expensive and time-consuming task. Here we propose a highly tunable, cost-effective, yet usable pipeline to predict enhancers. The implementation of our framework yielded a list of novel potential enhancers, as well as updated annotation data regarding cluster composition for all known olfaction-related sensory GPCR genes.

## Results

We investigated the mouse, rat and human genomes. After retrieving genomic coordinates for most receptor gene families mediating the sense of smell (that is, FPR, OR, TAAR and VR genes), we grouped them by species according to expression pattern and/or inferred gene function. Specifically, *MOE* was the name given to any list grouping OR and TAAR genes; *VNO* was the term used for groups of FPR and VR genes. Finally, we called *olfactome* any list produced by merging MOE and VNO lists — not to be confused with the olfactome introduced by Galizia et al. [61]. We used each of these grouped lists to produce a detailed classification of clusters and solitary genes.

For the identification of clusters and solitary genes one may first define a genomic distance above which two receptor genes are considered as belonging to different *loci*. This would specify how isolated has a gene to be so that it can be termed solitary and, analogously, how close must two genes be to say that they belong to a common structural unit, i.e. a cluster. We employed an ad hoc-built bioinformatic pipeline to characterize number and identity of clusters and solitary genes, taking as relevant genomic intervals (called *threshold* or *cutoff* values) spanning from 0.1 Mb to the whole chromosomal length of the widest chromosome found in each genome. As a comparison and solely for the mouse MOE list, we also tested a k-means clustering strategy.

**Fig. 1:**
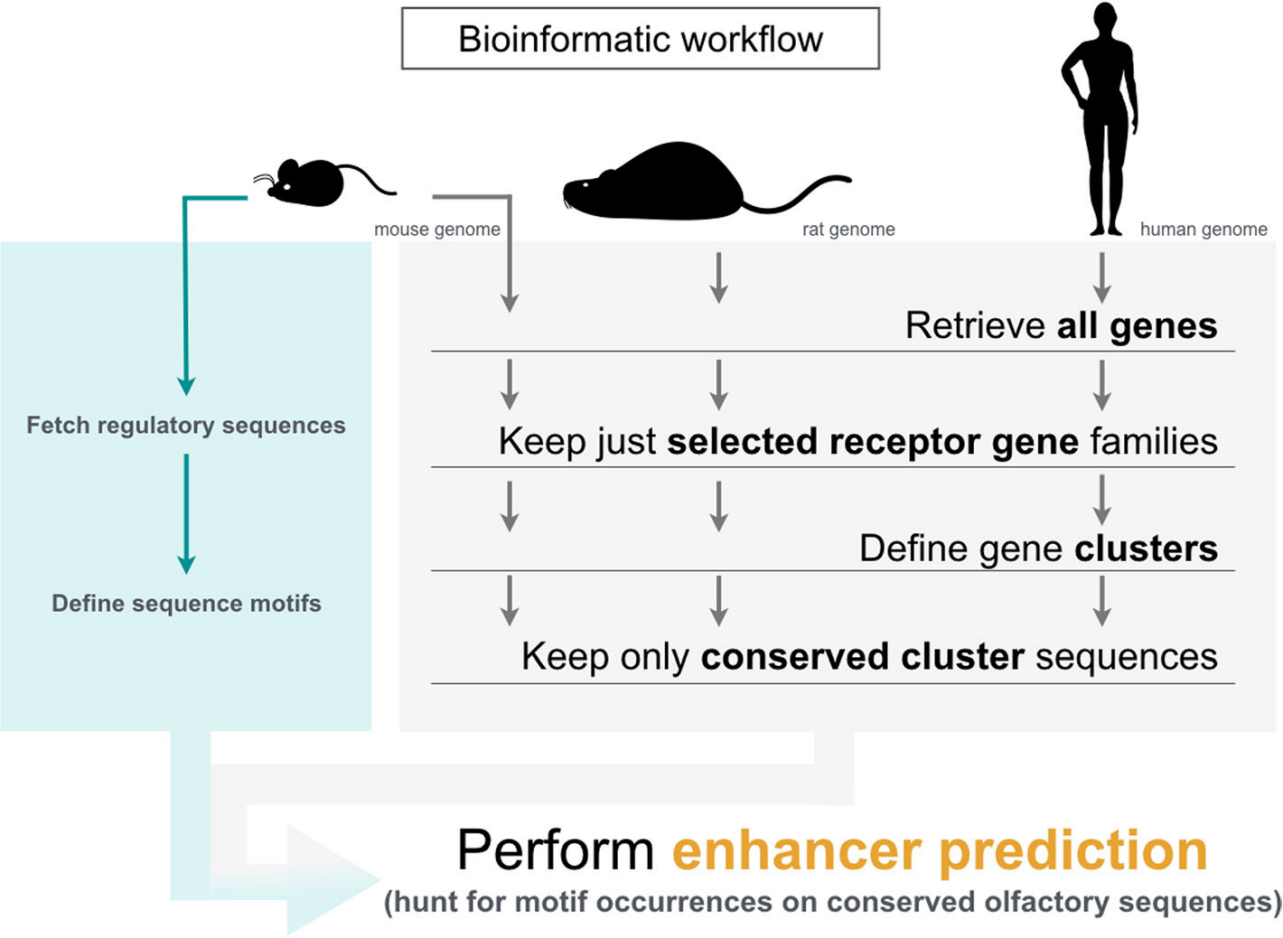
Outline of the bioinformatic framework. We retrieved gene information, including coordinates, for all genes annotated at Ensembl within the genomes of mouse (GRCm38), rat (Rnor_6.0) and human (GRCh38). From these, we considered most receptor genes responsible for the detection of odorants, i.e. odorant (OR), vomeronasal (VR), trace amine-associated (TAAR) and formyl peptide (FPR) receptor genes. For mouse only, T-cell receptor (TCR) genes were also kept. In every species, we combined gene families as such: OR and TAAR genes into a *main olfactory epithelium* (MOE) list; VR and FPR genes into a *vomeronasal organ* (VNO) list. MOE and VNO were also merged to form a list named *olfactome*. Members for each of the studied gene families are typically packed next to each other in a few chromosomal locations, forming clusters. We assessed number and identity of *loci* within each combined list (and for mouse TCR genes). Crucial for the definition of a *locus* is the adoption of a *threshold* (or *cutoff*) distance, above which two neighboring genes are considered as belonging to different chromosomal entities. Such value was varied between 0.1 Mb and the length of the widest chromosome found within its genome, each time yielding a specific *locus* composition. For selected cluster architectures, we fetched evolutionarily conserved sequences found within (or nearby) defined genomic locations; on these we predicted novel gene enhancers, based on sequence motifs mostly derived from known mouse OR promoters and regulatory elements

Apart from helping identifying clusters and solitary genes, the study served to make inferences regarding the putative presence of regulatory features among OR clusters, namely candidate elements. In addition, the pipeline works as a general tool to characterize the genomic architecture of other clustered gene families, whether they are involved in olfaction or not. Primarily as a proof of concept, we studied the genomic organization of TCR clusters for the mouse. Figure 1 outlines the key steps of our framework.

### Genomic architecture of the selected gene families

**Fig. 2:**
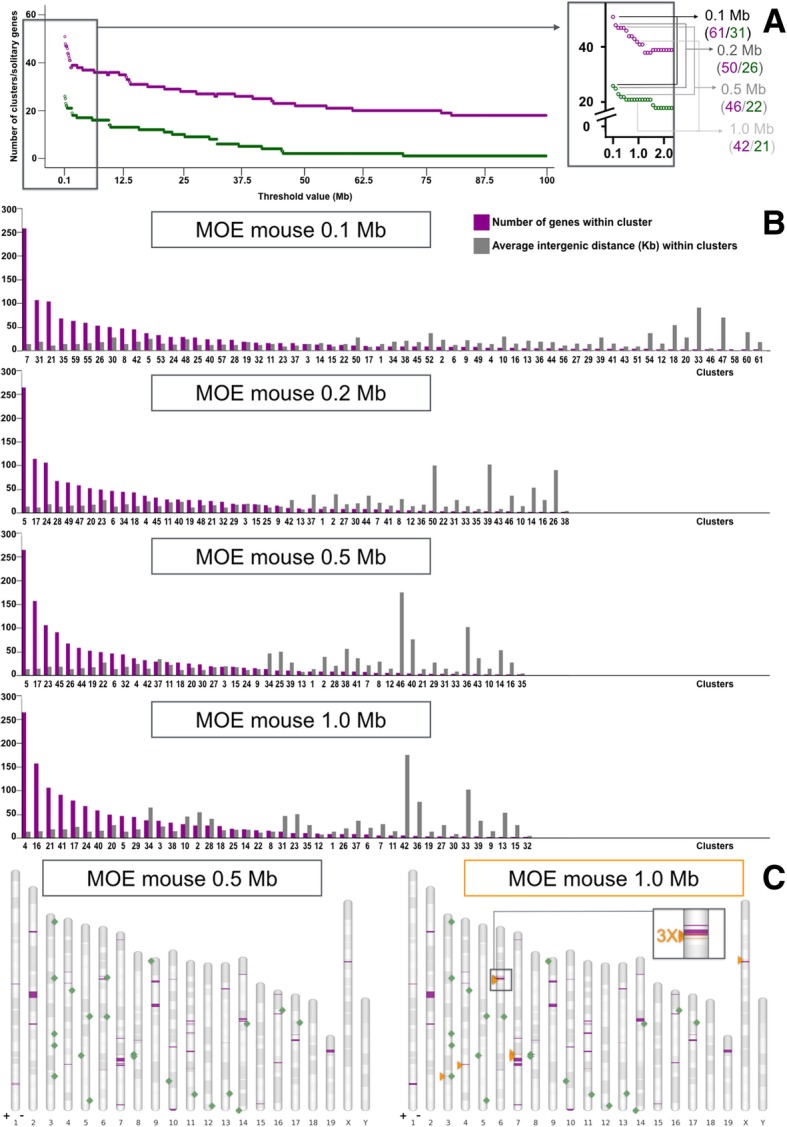
Olfactory receptor *loci* according to different cutoff intergenic distances. **a** Line chart reporting number of clusters (magenta) and solitary genes (green) of the mouse main olfactory epithelium (MOE) list, for threshold distances between 0.1 and 100 Mb (threshold increment = 0.1 Mb). The shadowed box (magnified on the right) details on cluster (magenta) and solitary gene (green) number for threshold values equal to 0.1, 0.2, 0.5 and 1 Mb. **b** Bar charts for architectures derived from the four aforementioned cutoff distances. Each couple of columns refers to a cluster: magenta bars indicate the number of MOE genes found in it, whereas gray bars report the average distance between its pairs of neighboring members. Clusters are numbered according to chromosomal location, but are presented (left to right) from the richest (in terms of number of genes) to *miniclusters* (i.e. systems made up by two genes). Bar charts do not display solitary genes. **c** Chromosome charts mapping clusters (magenta intervals) and solitary genes (green squares) for the mouse MOE 0.5 (left graph) and 1 Mb (right graph) threshold. The latter is one of those lists utilized to search for elements (we indicated that by enclosing its title line in an orange box). Predicted enhancers are depicted as orange triangles. While elements are invariably presented as located on genomic plus (+) strand, solitary genes are annotated on their sense strand (be it plus or minus, −). A location containing three predicted elements close to each other is magnified (black shadowed box). Chromosome bands represent Giemsa staining

**Fig. 3:**
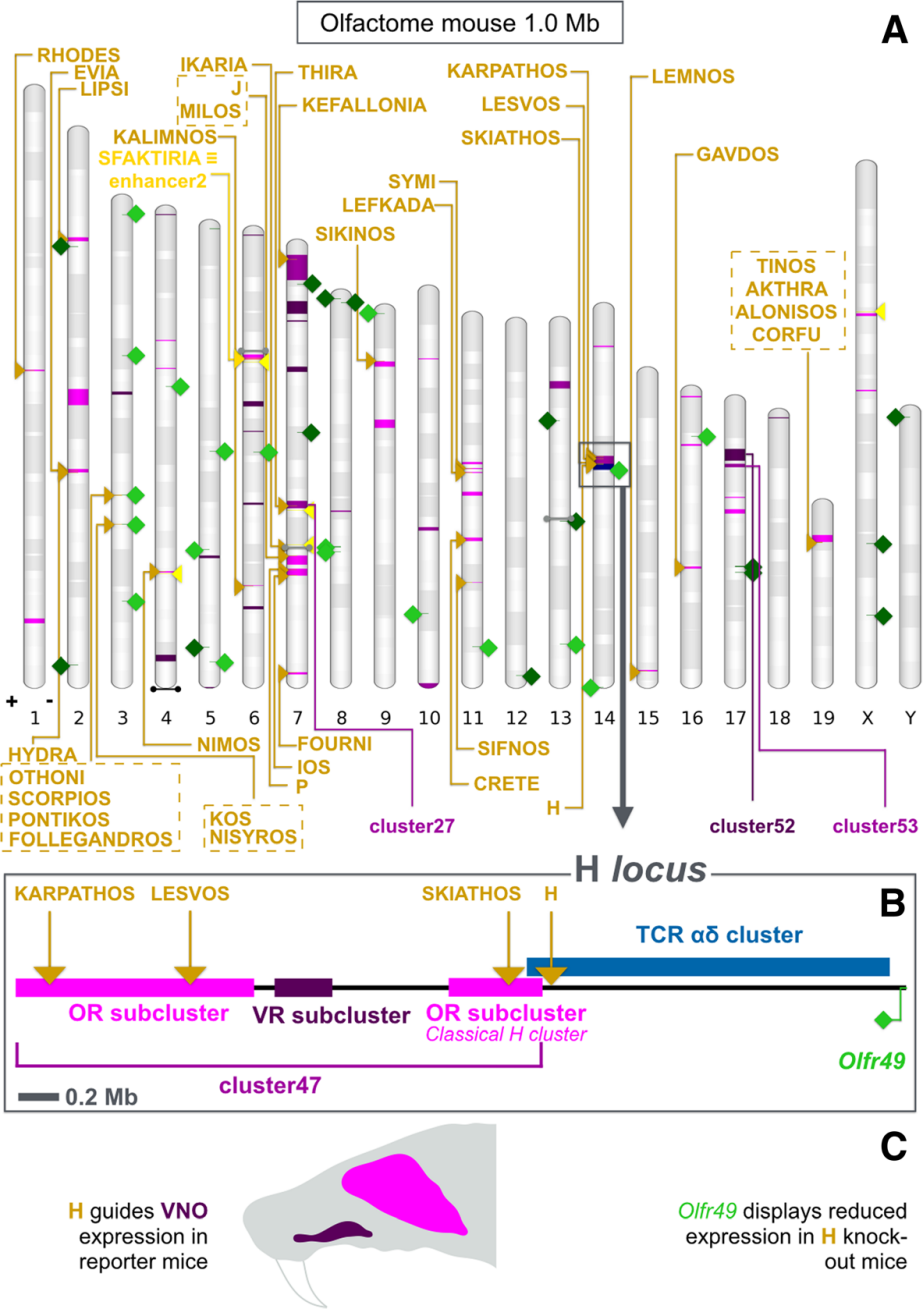
The mouse olfactome. **a** Chromosome chart for the mouse olfactome 1 Mb threshold. Magenta intervals mark locations for clusters composed by three or more genes: light magenta for odorant receptor (OR) and/or trace amine-associated receptor-only clusters, dark magenta for pure vomeronasal receptor (VR) and/or formyl peptide receptor clusters, mid magenta for those containing members from both groups. Names for a few notable clusters are explicitly indicated. Miniclusters are reported as dumbbells, in light gray if composed solely by OR genes, black if made up by just VR genes, and dark gray when mixed (with the same meaning as for regular clusters). Solitary genes are rendered as green squares, depicted on the genomic strand that contains their sense strand; more specifically, dark green indicates solitary VR genes, light green is used for solitary OR genes. Previously discovered/hypothesized (dark yellow) or newly predicted (light yellow) elements are reported as triangles (on plus strand, +, unless otherwise required due to graphical constrains). Known enhancer names are noted down. Names for our candidate elements are not explicitly reported, but these are simply numbered according to chromosomal location. Putative enhancer2 overlaps extensively with Sfaktiria. Near to H, a blue interval corresponds to the αδ T-cell receptor (TCR) cluster. Chromosome bands represent Giemsa staining. **b** Detail on the H *locus* (magnified from panel A, shadowed box). Considering OR and VR genes together (here as cluster47) causes two OR groups (one being the classical H cluster) to be united by a bridge of VR genes; on the right, the aforementioned TCR cluster is followed by the solitary OR gene *Olfr49*. **c** H *locus* as a possible functional olfactome unit. While an effect of H on VR gene regulation awaits to be proven, H drives vomeronasal organ (VNO) expression in transgenic reporter mouse strains. The solitary gene *Olfr49* has been reported as mildly affected by H deletion. Based on its genomic isolation, this effect has been considered to be a mere consequence of strain variations between H-deficient and control mice

List of genes for each of the selected gene families were obtained (Table 1). At 1 Mb threshold, for OR and TAAR genes (MOE list including pseudogenes), 42 clusters and seven intact solitary genes are identified (*Olfr19*, *Olfr49*, *Olfr266*, *Olfr267*, *Olfr370*, *Olfr371*, *Olfr1402*). Among all clusters there are four *miniclusters*, as we call clusters composed by a single pair of genes: cluster9 (pair *Olfr460*-*Olfr461*, with an intergenic distance of 14 Kb and oriented tail to tail), cluster13 (pair *Olfr290*-*Olfr291*, having an intergenic distance of 53 Kb and oriented head to tail), cluster15 (pair *Olfr520*-*Olfr521*, separated from each other by 27 Kb and oriented head to tail), and cluster32 (pair *Olfr465-ps1*-*Olfr466*, that has an intergenic distance of just 4 Kb and is also oriented head to tail). Additional file 8: Table S1 contains detailed information about *loci* for some selected lists; a more comprehensive report is found in Additional file 3. The mouse MOE 1 Mb cutoff architecture has an average intergenic distance of ~ 43 Kb. Mean distances between neighboring genes within single clusters of the list vary from 4 to 175 Kb, respectively for the aforementioned cluster32 and for cluster42. Figure 2 shows data regarding cluster/solitary gene number at various threshold values for the mouse MOE, with a focus on cluster composition for some chosen values; Fig. 3 is dedicated to the mouse olfactome. Additional file 4 contains custom annotation tracks for clusters and solitary genes for most relevant genomic architectures; Additional file 1: Figure S1 shows the differences between the genomic architecture obtained for the mouse MOE using our distance-based clustering method (1 Mb threshold) compared with a k-mean clustering approach.

**Table 1:** Number of genes for the selected gene families in each of the studied genomes

Species	OR genes (whole)	OR genes (intact)	VR genes (whole)	VR genes (intact)	TAAR genes	FPR genes	TCR genes
Mouse	1364	1109	548	330	16	7	269
Rat	1363	1352	173	171	17	6	–
Human	779	404	63	3	9	3	–

Gene families are odorant receptor (OR) genes, vomeronasal receptor (VR) genes, trace amine-associated receptor (TAAR) genes, formyl peptide receptor (FPR) genes and T-cell receptor (TCR) genes (just for the mouse)

### Predicted elements around defined *loci*

Aiming to enrich in elements (cf. [26, 42]), we fetched evolutionarily constrained sequences found within or nearby obtained *loci* for some of our genomic architectures (Additional file 5). Pairwise alignments of known enhancers against this newly defined genomic portions confirmed that a subset (about 1/3 for mouse MOE 1 Mb cutoff) of known enhancers is retained.

**Fig. 4:**
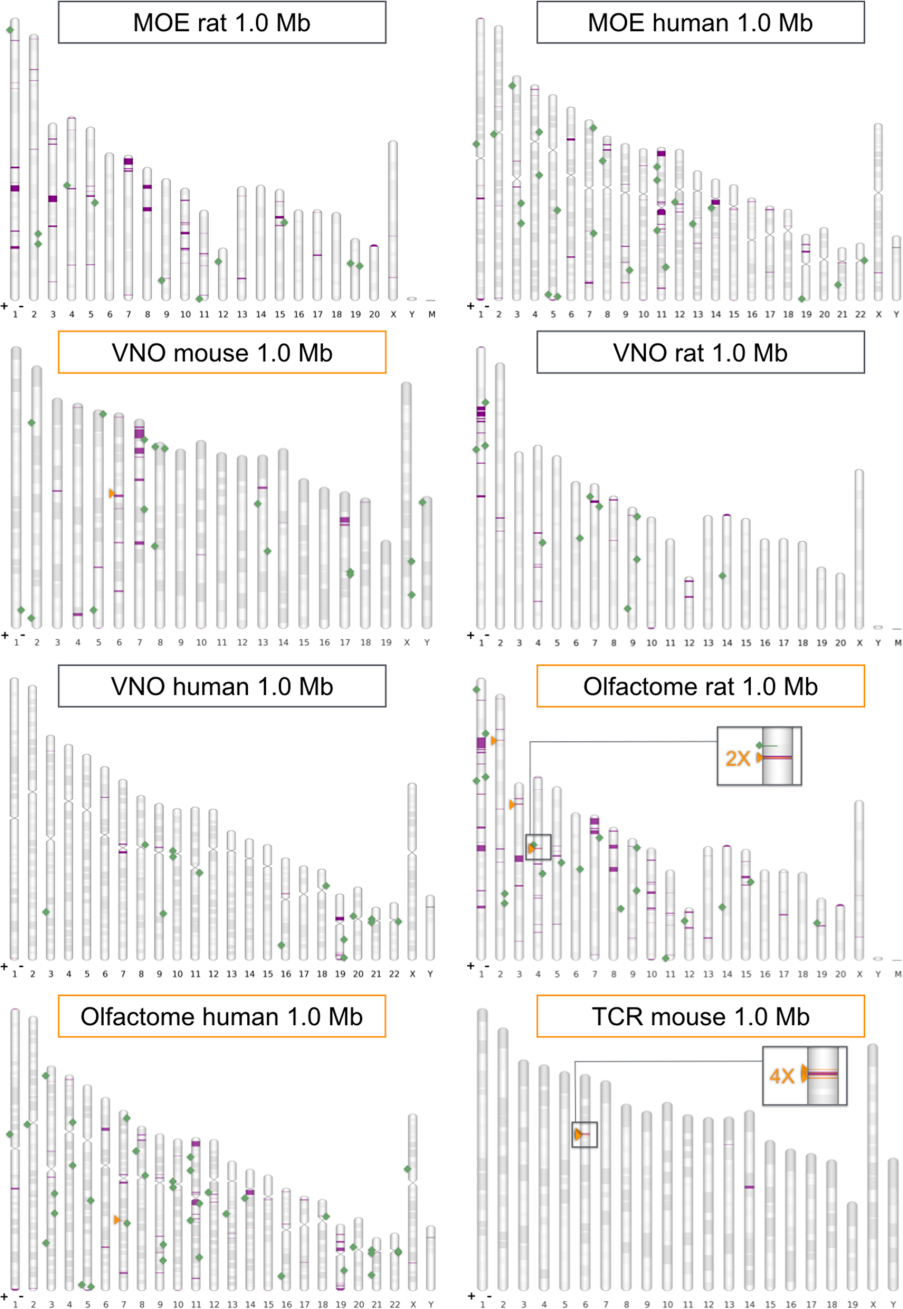
Genomic architecture for groups of clustered receptor genes. Selected chromosome charts mapping clusters (magenta intervals) and solitary genes (green squares); list types are main olfactory epithelium (MOE), vomeronasal organ (VNO), olfactome or T-cell receptor (TCR) genes. Solitary genes are depicted on their sense strand (on genomic plus or minus filament, + or -). For those lists used for element prediction (their title line is boxed in orange) we also report candidate enhancers (as orange triangles, always on genomic plus strand). When putative elements are found to be in close proximity, (shadowed) magnified boxes are provided. Chromosome bands represent Giemsa staining

**Fig. 5:**
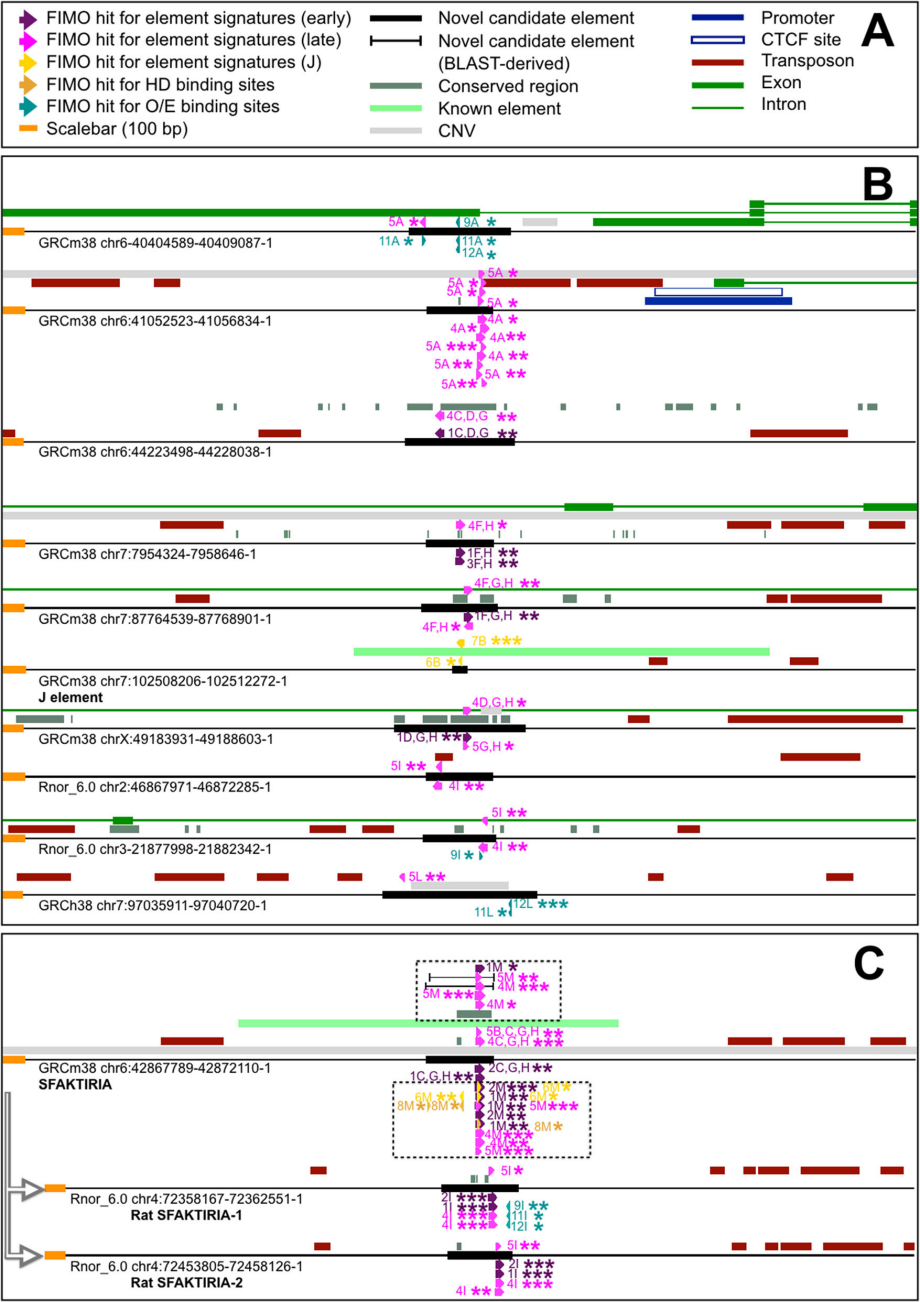
Selected candidate elements in the three studied genomes. **a** Graphical legend for sequence features present in panel B and C. FIMO hits for position-specific weight matrices (PSWMs) are reported as 3′-pointing arrows. Occurrences for matrices 1 to 3 are labeled as “FIMO hit for element signatures (early)”, while those for PSWMs 4 and 5 are named “FIMO hit for element signatures (late)”; hits for matrices 6 and 7 are indicated as “FIMO hit for element signatures (J)”. Finally, occurrences for single transcription factor binding sites are called “FIMO hit for HD binding sites” (for homeodomain sites, PSWM 8) and “FIMO hit for O/E binding sites” (for olfactory/early B sites, PSWMs 9 to 12). We named “Novel candidate element” any region (derived from conservation tracks and used for FIMO searches) yielding FIMO hits for two rounds of PSWM alignment. “Novel candidate element (BLAST-derived)” sequences are produced by a pipeline variant, which requires conserved stretches to contain a core 13-bp motif prior to undergo FIMO analyses. **b** DNA maps for ten selected predicted elements (extended 2 Kb upstream and downstream) in mouse (GRCm38), rat (Rnor_6.0) and human (GRCh38). Each putative enhancer region (genomic coordinates on the left) is reported as a black thin arrow encompassing the whole page width. On it, annotations are found. Each FIMO hit comes with a number (corresponding to the PSWM associated to it), a significance level (* for q < 0.05; ** for q < 0.01; *** for q < 0.001) and one or more capital letters. These indicate the list(s) in which the candidate element was found; for mouse, 1 Mb threshold: A, T-cell receptor (TCR) genes; B, main olfactory epithelium (MOE), BLAST-derived pipeline variant; C, MOE; D, MOE (pseudogene-flagged genes pruned); E, vomeronasal organ (VNO); F, VNO (pseudogene-flagged genes pruned); G, olfactome; H, olfactome (pseudogene-flagged genes pruned); for rat and human, respectively (also 1 Mb threshold): I, olfactome; L, olfactome. FIMO hits resulting from a first-round PSWM alignment are represented above the element on which they are found; those yielded by second-round FIMO analyses are depicted below (second-round occurrences identical to first-round ones are systematically present but not reported). Within each round, when significance level varies between lists asterisk number conforms to its maximal value. **c** Sfaktiria is independently found by our framework, and it is duplicated in the rat. Maps are rendered as in panel B. Dashed shadowed boxes on mouse Sfaktiria highlight features deriving from the aforementioned pipeline variant, including first- and second-round FIMO hits; these comprise PSWM (1, 2, 6, 8) occurrences that do not contain information originating from Sfaktiria itself. Sfaktiria has two nearly identical counterparts in rat (at least in Rnor_6.0), pointed by gray-bordered shadowed arrows on the left

Based on conserved sequence stretches found on known elements/OR promoters and therefore expected to contain key regulatory features, we designed a set of position-specific weight matrices (PSWMs). Twelve PSWMs were selected in total: three of them (numbered 1, 2 and 3) were mostly based on essential sequences found on H and P elements; these covered known HD and O/E TSBSs, including a crucially important 13-bp-long motif known to be identical between the two enhancers, cf. [62]. PSWMs 4 and 5 contain typical element signatures for class II OR genes, i.e. variations of the same core motif found on H and P. Matrices 6 and 7 contain signatures, in different Muridae *taxa*, for the only known enhancer regulating class I OR genes (J); the remaining PSWMs focus on single kinds of TFBSs found on elements/OR promoters, either HD (matrix 8, actually identical to the one presented in [25]) or O/E sites (matrices 9, 10, 11 and 12). Graphical representations of the PSWMs are found in Additional file 2: Figure S2, whereas the matrices themselves are provided as Additional file 6. To various degrees, these twelve motifs represent element hallmarks. In our experience, matrices featuring TFBSs for HDs are more informative than those built solely on O/E sites, so we considered PSWMs 1 to 8 to be more relevant, and dubbed them *core* matrices. We screened our datasets of evolutionarily conserved sequences for occurrences of regions with significant similarity to each of our twelve motifs. Ranges displaying at least a supported hit were retained for a second-round examination, again performed against all the twelve PSWMs; eventually, only those stretches containing first-round hits for one or more core matrices were kept. Selected candidates are annotated on chromosome maps in Figs. 3 and 4, together with the cluster/solitary gene architecture used to identify them. Predicted elements are also found as annotation tracks in Additional file 7. Lastly, best putative elements are presented as maps in Fig. 5.

Primarily to show that the procedure can yield known elements in absence of *circuli in probando*, we pairwise-aligned the aforementioned 13-bp-long motif against all conserved stretches within the mouse MOE 1 Mb cutoff *loci*. Best-aligning sequences were screened for PSWM hits, using those matrices that do not include information derived from [28, 42]. Most robust occurrences for PSWM 1 and 2 overlap with Sfaktiria for more than 300 bp, at chr6:42869789–42,870,110. Apart from Sfaktira, the method yields some novel potential elements. These results are summarized in Table 2.

**Table 2:** Mouse elements yielded by a variant of the procedure that exploits only information derived from H and P

Sequence set of origin	mm10 coordinates	PSWMs with at least a significant hit (first round)	PSWMs with at least a significant hit (second round)	Notes
EPO/mouse MOE 1 Mb threshold	chr19:14694517–14,694,929	1, 4, 6, 8	1, 3, 4, 6, 8	Predicted enhancer in close proximity
EPO/mouse MOE 1 Mb threshold	chr14:49188564–49,189,030	1, 4, 6, 8	1, 2, 4, 5, 6, 8	On a retained intron
EPO/mouse MOE 1 Mb threshold	chr11:107760321–107,760,529	1, 8	1, 4, 6, 8	–
EPO/mouse MOE 1 Mb threshold	chr7:100639235–100,639,627	1	1, 5, 8	–
GERP/mouse MOE 1 Mb threshold	chr6:42869807–42,870,110	1, 2, 4, 5	1, 2, 4, 5, 6, 8	Overlaps with Sfaktiria
GERP/mouse MOE 1 Mb threshold	chr6:42869789–42,870,106	1, 2, 4, 5	1, 2, 4, 5, 6, 8	Overlaps with Sfaktiria

Sets of evolutionarily conserved mouse sequences found within olfactory *loci* were screened for occurrences of position-specific weight matrices (PSWMs) obtained from multi-aligments of H and P-related sequences (no other known elements being included). Such sets were obtained intersecting the mouse MOE 1 Mb threshold list with mouse Ensembl tracks as either “36 eutherian mammals EPO low coverage” (EPO/mouse MOE 1 Mb threshold) or “GERP constrained elements” (GERP/mouse MOE 1 Mb threshold)

## Discussion

### Effects of genome annotation quality and cutoff values

The present work is based on gene features available in public repositories. As such, the method is highly dependent on the quality of genomes and their annotation. While the species we selected are among the best known *taxa* in that perspective (certainly the most characterized mammals), we recommend special care when trying to adopt analogous strategies to poorly annotated genomes: it is easy to note, for instance, that missing (i.e. unannotated) genes produce gaps that artificially inflate number of clusters and solitary genes.

To provide some terms of comparison, the number of mouse intact OR genes found at Ensembl and those categorized by Niimura et al. [16] are in good agreement (respectively, 1109 vs. 1130), and the same can be claimed for human OR genes (amounting to, respectively, 404 and 396). For the rat, Ensembl tracks seem to overestimate the number of intact genes (1352 against 1207 for Niimura et al.), probably at the expenses of pseudogenes/truncated genes (nine compared to 570, respectively). We mitigated errors deriving from possible misattribution of intact state to non-functional genes by forking our procedure, so that main investigations were conducted both with and without taking into account pseudogene-flagged entries.

Artifacts deriving from unannotated genes would likely be observed more often at especially low threshold values, perhaps being less noticeable at 1 Mb cutoff value. Such distance, the one we adopted for in-depth analyses, is in line with existing standards in the field (e.g. [23, 25]), and roughly accommodates the central range of *cis* activity of known elements, cf. [27]. The value is also proximal to the flex of the curve that illustrates the effects of threshold changes on the number of mouse MOE clusters (Fig. 2), meaning that 1 Mb is close to a span above which cutoff adjustments have less impact on the number of cluster.

Imposing a threshold distance is only one out of several possibilities to define clusters and solitary genes. However, it should be kept in mind that the ultimate scope of studying gene clustering in olfaction is the understanding of local regulatory dynamics. When we tried to cluster genes from the mouse MOE list by means of k-means clustering (Additional file 1: Figure S1), we observed two unwanted phenomena: the first one is an oversplitting of regions with high gene density (e.g. in chromosome 7); the second is the creation of broad clusters that encompass most of the chromosomal length (e.g. in chromosome 3). Our method does not seek to maximize homogeneity among intergenic distances within a cluster, rather it attempts to extract biologically relevant information. While it is clearly not reasonable to think of a one-size-fits-all threshold as a way to systematically tell apart *loci* as functional units, there are few doubts that the use of predefined cutoffs is common practice in high-throughput studies. Each architecture provided by a single cutoff value should be intended, strictly speaking, as a conventional entity merely based on structure. Nevertheless, different architectures will enrich for different functional features. In the mouse genome, for instance, using a threshold of 10 Mb we obtain just a few solitary OR genes, each having very little chances of sharing *in cis* regulation with other OR genes. Instead, moving the cutoff value to 0.1 Mb produces a higher number of solitary OR genes, all with a much increased probability of having regulatory features in common with adjacent OR genes.

### Organizing clusters to understand them

Varying the way genes are grouped obviously produces different *locus* architectures: each list brings attention to specific chromosomal locations. Cluster32 (chr13:65138089–65,156,152) of the mouse MOE 1 Mb threshold list is a minicluster: it contains the newly annotated pseudogene *Olfr465-ps1*, neighboring *Olfr466* (previously regarded as the most isolated OR gene of the mouse genome [25]). The corresponding list devoid of pseudogenes defines cluster35 (chr16:3591042–3,844,747) as a binary entity that comprises the model gene *Olfr15* (*MOR256–17*) — a strongly expressed [63], broadly tuned and well characterized OR gene [64, 65]. Were its promoter or the one of its companion *Olfr161* knocked out, a variation on the expression level/OSN counts for the other gene of the pair would highly suggest the presence of a close by regulatory element. In the mouse olfactome 1 Mb cutoff, cluster27 (chr7:84810428–87,037,968) harbors an otherwise OR minicluster surrounded by VR genes, the pair *Olfr291*-*Olfr290* at chr7:84853553–84,920,861; cluster47 (chr14:49894258–52,495,749) is an extended version of the H cluster, made up by two blocks of OR genes linked by three VR genes. Downstream to the cluster there is H, then the αδ TCR cluster, and finally the solitary gene *Olfr49*, separated by the last gene of cluster47 by 1.7 Mb. We suggest that this all range, or a relevant part of it, might at least be evaluated as a functional unit (Fig. 3). In fact, even in lack of formal evidence supporting a role for H in VR regulation, H reporter mice do display staining in the VNO (cf. panel 2A by Markenscoff-Papadimitriou et al. [28]). A mild downregulation of *Olfr49* following H deletion was observed by Khan et al. [27], who cautiously interpret this result as a distortion related to the comparison of mice with slightly different genetic backgrounds, but a direct action of H on *Olfr49* cannot still be ruled out. Lastly, it is well established that TCR clusters are invariably found in close linkage with OR *loci*, and that the two gene families share a number of regulatory peculiarities, cf. [32, 59]. The validation of this idea would require a quantitative evaluation of the expression for some more OR, VR and TCR genes of the region in MOE, VNO and T lymphocytes, both in wild-type and H-deficient mice. Other notable entities of the mouse olfactome 1 Mb threshold are cluster52 (chr17:17703941–21,323,766), which contains all FPR genes interspersed within VR genes, and cluster53 (chr17:22547941–23,492,471), a VR cluster ending with a single, otherwise solitary OR pseudogene (*Olfr752-ps1*).

Within the rat olfactome 1 Mb cutoff, we highlight cluster3 (chr1:59765835–70,496,347), mainly a VR cluster that contains a few FPR-like and OR genes; then there is cluster7 (chr1:116152667–116,885,644), a FPR/VR-mixed minicluster formed by *Fpr3* and *Vom2r25*. Finally, we mention cluster45 (chr9:101222846–101,269,843) and cluster69 (chr19:27534854–28,378,118), two OR/VR-mixed miniclusters respectively composed by the pairs *Vom1r64*-*Olr178* and *Olr1666*-*Vom1r21*. *Olr1666* is orthologous to the mouse solitary OR gene *Olfr371* [16, 25], but considering OR and VR clusters as a single unit causes the gene not to be solitary anymore. It is intriguing to think at cluster69 as a possible functional unit: *Olfr371* is in fact expressed in the VNO of newborn mice by RNA-Seq (EMBL-EBI ArrayExpress [66], accession number E-ERAD-169).

For the human genome, cluster34 (chr11:54591480–59,749,574) is the richest cluster of the olfactome 1 Mb cutoff list; it harbors a single VR pseudogene (*VN2R9P*) in between OR genes. Cluster15 (chr6:131700469–132,646,003) of the same list is the TAAR *locus*, which starts with a single, otherwise remarkably isolated (39 Mb) OR gene: *OR2A4* [25].

Our study probes with unprecedented detail the genomic arrangement of chemosensory genes responsible for the sense of smell. This is especially true for multifamilies other than OR genes, like VR genes. OR and related chemosensory genes for smell are abundant, and their clusters have a deep functional meaning. There is a strong chromosomal association for some non-OR gene receptor families to OR *loci*. In such a complex scenario, organizing gene entries in the most convenient way may become an important step in deciphering the molecular dynamics that underlie OR gene choice. Also, evaluating multiple chemoreceptor gene types at a time is a new approach in the olfaction field, and hopefully such strategy will facilitate future analyses of functional synergies.

In addition to conventional clusters and solitary genes, we considered miniclusters as possible new models for the study of the one-neuron one-receptor rule. In fact, while enhancers can certainly exist around solitary genes, defining an element as a sequence regulating the probability of choice of an OR gene within a defined *locus* (e.g. [27]) implies at least two nearby OR genes for an element to exist. Miniclusters are the simplest arrangements for which a dedicated element can carry out a process of stochastic choice.

### Element signatures and novel candidate elements

A critical review of the literature suggests that OR enhancers are mostly unknown. A recent study [60] proposes 63 elements, about a third more than just three years before; 78 have been hypothesized so far in total [26–28, 37, 41, 42, 60]. Out of four elements confirmed with knock-out mice, one was discovered only very recently [42]. With merely a handful of OR genes being strongly down-regulated each time a loss-of-function mutation is produced, we should expect the total number of OR enhancers to be in the order of hundreds, cf. [27]. If even half of the elements proposed to date proved to be real, the unknown ones would still constitute a majority.

Here we present thirty predicted elements that passed strict selection criteria: they are found within or close to *loci* of a given architecture track, they are evolutionarily conserved and possess statistically significant resemblance with known elements. Among the 26 candidates obtained from the main procedure, i.e. excepting locations exclusively shown in Table 2, 21 are mouse sequences. Many of them — depending on genomic context — are expected to be OR enhancers, but some might also or even mainly regulate other chemosensory receptors, like VR genes. Instances of possible non-strictly-OR elements are highlighted in the olfactome 1 Mb cutoff. Its OR/VR-mixed cluster27 neighbors both a novel candidate enhancer (enhancer4 of the aforementioned list, chr6:44225498–44,226,038) as well as the putative element Thira [28]. These two could in principle regulate both VR and OR genes found in the *locus*. Similarly, H might control the expression of a few VR genes. Four elements are predicted for the TCR list, near to the ß TCR cluster (categorized as enhancer1, chr6:40406589–40,407,087; enhancer2, chr6:41054523–41,054,834; enhancer3, chr6:41698734–41,699,042; enhancer4, chr6:42268798–42,269,136). VNO 1 Mb cutoff lists, with and without including entries annotated as pseudogenes, display a total of seven candidate enhancers, of which three neighbor no olfaction-related chemosensory genes other than VR genes. One of them (sequence chr5:150744011–150,744,410) is adjacent to the *locus* of *Vmn2r18*, a three-gene system that comprises also two VR members flagged as pseudogenes.

Enhancer1 (chr3:132160901–132,161,239) of the MOE 1 Mb threshold list is notable for being next to the solitary OR pseudogene *Olfr375-ps1*. Other predicted mouse elements probably worth of mention are sequences found at chr6:44225498–44,226,038 and chr7:87766539–87,766,901 (simply because they are robust candidates), as well as the range chrX:49185931–49,186,603, located on a sex chromosome (the only other putative element in the X chromosome being Schoinousa [60]). Finally, chr7:102510206–102,510,272-1 is J; yet this output should not be considered an self-supporting rediscovery, as PSWMs providing hits on the range were derived from J itself.

Final confirmation for elements requires their genetic ablation. However, our framework identifies Sfaktiria as a strong hypothetical element even when matrices based solely on H and P are used. It is highly implausible for such result to occur by chance, so we believe the independent detection of Sfaktiria constitutes per se a first validation of our method.

Our work mainly focuses on mouse. Rat and human enhancers were searched only within the respective olfactome 1 Mb threshold lists, and element signatures are motifs discovered in mouse. For the rat, we still found a total of four candidates: the first two have currently no mouse counterparts, and are located at chr2:46869971–46,870,285 and chr3:21879998–21,880,531. The remaining pair (chr4:72360167–72,360,551, chr4:72455805–72,456,126) is highly homologous to mouse Sfaktiria, and resides on an OR region that appears to be duplicated in the rat. This is reminiscent of the mouse P being remarkably similar to the nearby promoter of *Olfr713* (better known as *P3*) [41], cf. [62]; if confirmed, the two Sfaktiria-like rat sequences would constitute a blatant example of how new elements can evolve when segmental duplications occur within OR clusters. Consistent with its higher taxonomic distance from the mouse, the only element predicted by our procedure for the human is range chr7:97037911–97,038,720, situated upstream to cluster18 (chr7:97946987–97,967,074), virtually a completely pseudogenized minicluster.

Element signatures are likely pivotal sequence features for OR gene choice. Recently they were found to be diffusely required for OR expression [60]. By the time more elements are known, it would be unsurprising that such signatures become increasingly relevant tools to hunt for new enhancers; with refined matrices, pipelines akin to the one proposed here might flourish.

## Conclusions

As genomic architecture has a primary role for OR clusters, chromosomal linkage of non-OR gene families to OR genes is worth close attention. This is especially true for the vast majority of other chemosensory receptor genes mediating olfaction, which share key regulatory aspects with OR genes, e.g. [46]. Notably, to the best of our knowledge nobody has ever searched for VR enhancers or checked for VR regulatory activity from known OR enhancers. The result is a rampant discrepancy between the major efforts towards the discovery of new elements for OR genes and the virtually complete disregard for possible VR enhancers. At least understanding whether known OR elements (notably H) exert some control over VR gene expression would be relatively uncomplicated. Similarly, other potential synergies with non-olfactory genes, as immune receptor genes, might be promptly evaluated. The method we propose can be easily adapted to different groupings of gene types, and the data regarding *locus* composition of various receptor genes transducing olfactory signals will hopefully facilitate further studies across such gene families.

In addition to regular clusters and solitary genes, we introduce the concept of minicluster: these are interesting systems for the investigation of the mechanisms in behind the one-neuron one-receptor rule, as they constitute the most elementary configuration that — by definition — can harbor a *locus* control region that promotes a local random process of gene choice.

Contrary to common statements, primary sequence contains enough information to predict at least some OR elements (strictly speaking, Sfaktiria). While certainly epigenetic markings can also be taken into account, here we want to stress the relevance of sequence signatures as element hallmarks that hold potential as means for the discovery of new enhancers. The current work adds thirty candidates to the set of putative elements proposed to date. The definition of novel regulatory regions ultimately involves the generation of genetic evidence, which typically requires extensive in vivo validation of all possible enhancers, including ours. But our study demonstrates a straightforward approach that might at least guide future genetic analyses. With so much work ahead in the search for elements, preparatory screening procedures are likely a necessary step that requires careful optimization.

## Methods

### Species and genomes

Our study considers three mammalian genomes, the house mouse (*Mus musculus*, strain C57BL/6 J) assembly GRCm38.p5, the brown rat (*Rattus norvegicus*, mixed strain) assembly Rnor_6.0 and the human (*Homo sapiens*) assembly GRCh38.p10. Species choice was based on genome quality, with special attention towards annotation; while mouse is vastly recognized as a model for mammalian genetics, a second selection criterion for rat and human was their increasing taxonomic distance from the mouse (respectively, same family, Muridae; same superorder, Euarchontoglires).

### Gene retrieval

For each of the three genomes considered, we downloaded genomic coordinates for all genes via BioMart [67] at Ensembl [68]. After pruning those entries residing on alternative scaffolds, we prepared single lists for selected gene families, i.e. FPRs, ORs, TAARs, TCRs and VRs; these were then sorted according to chromosomal location. Sorted lists were also combined according to functional relevance: those gene families mainly devoted to sensing within the MOE (OR and TAAR genes) were merged in a *MOE* list; those mostly providing sensory receptors for the VNO (FPR and VR genes) were unified as a *VNO* list. Finally, MOE and VNO lists were further combined to form *olfactome* lists. Gene sets devoid of entries flagged as pseudogenes were also prepared. Lists eventually considered for downstream analyses, each with or without pseudogenes, were: MOE, VNO, TCR and olfactome for the mouse; MOE, VNO, and olfactome for the rat; MOE, VNO, and olfactome for the human.

### Classification of olfactory and TCR *loci*

The genomic architecture of every set was thoroughly assessed via local scripting: in order to do that, we first needed to define a *cutoff* genomic distance above which two neighboring genes were considered as belonging to different *loci*; such *threshold* was used for determining number and identity of both clusters and solitary genes. The process was iterated for cutoff values spanning from 0.1 Mb to the whole length of the widest chromosome of the genome, using a 0.1 Mb distance as increment. Clusters were numbered according to chromosomal location. We prepared BED-formatted feature track files for threshold values equal to 0.1, 0.2, 0.5 and 1 Mb.

To provide a term of comparison with our distance-based clustering strategy, we tested Ckmeans.1d.dp on the mouse MOE gene list; Ckmeans.1d.dp is a k-means clustering method for one-dimensional data [69]. We first split such list to obtain single-chromosome files; start and end positions of each gene were collapsed to yield a single central chromosome coordinate. Using the Bayesian information criterion, the algorithm identified an ideal number of *loci* for each chromosome, and then clustered genes in a corresponding number of sets. Single-chromosome output files were merged to obtain a k-means-based genomic architecture for the mouse MOE.

### Obtaining conserved sequences within identified *loci*

Clusters/solitary genes coordinates were expanded by 1 Mb at both ends. Evolutionarily conserved sequences within expanded ranges were then fetched by intersecting (via BedTools [70]) BED files with Ensembl tracks containing GERP constrained elements, i.e. regions identified by GERP as being under purifying selection [71–74]. Intersected ranges were broadened, upstream and downstream, by 150 bp. FASTA files for the yielded genomic intervals were obtained with UCSC Table Browser [75]. As a quality control, we retrieved FASTA sequences for all known elements, and systematically BLASTed [76] them against each of the FASTA files obtained.

The expanded mouse cluster/solitary gene MOE 1 Mb cutoff list was also intersected with the Ensembl track “36 eutherian mammals EPO low coverage” [77, 78]. The track annotates genomic regions conserved among Mammalia Eutheria. A FASTA file for these intersected ranges was generated, again via UCSC Table Browser.

### Preparing position-specific weight matrices

We retrieved FASTA sequences for all known elements, plus a few OR promoters. All these sequences came from C57BL/6 J, but when some enrichment was desirable orthologous regions from rat or other *Mus* species were retrieved via BLAT at Ensembl [79]. Multi-aligments (on T-Coffee [80] at MacVector [81]) of subsets of such strings were used to identify conserved stretches. By grouping such portions into different multi-FASTA files, we finally selected a set of PSWMs (via MEME Suite’s MEME [82, 83]), most of them representing core element motifs.

### Enhancer prediction

Using FIMO [84] from the MEME Suite, we scanned every FASTA file containing conserved regions within clusters/solitary gene *loci* for occurrences of each PSWM. FIMO converts log-odds scores into *p*-values by using a dynamic programming algorithm, which assumes a zero-order background model. Following the Benjamini and Hochberg procedure, p-values for every hit are converted to q-values, defined as the minimal false discovery rate at which a given occurrence is considered significant. α threshold for p-value and q-value significance was set to 0.05. Sequences with at least one significant PSWM occurrence were kept for a second round of FIMO analyses (again for all matrices, with an α value of 0.05). Those stretches harboring first-round hits for any of the core PSWMs were accepted as putative elements. Among them, detailed maps were prepared for selected candidates. BLAST searches were performed to find out whether known elements were found by the framework, and to figure out possible occurrences of a single element in more than one species.

A variant of the pipeline described above was implemented mainly to demonstrate the validity of the approach: we thought to use sequence information available before Markenscoff-Papadimitriou et al. [28] to see whether we could independently predict elements proposed within or after Markenscoff-Papadimitriou et al. [28]. There is a 13-bp-long (5′-TCATTAAAAAGTT-3′) perfect match among H, P and the promoter of *Olfr713*; we used this stretch as a query for BLAST searches on a couple of our FASTA lists of evolutionarily conserved ranges. More precisely, BLAST databases for this search were the one derived from the 150-bp-expanded intersection of the mouse MOE 1 Mb with the mouse GERP track, as well as that obtained intersecting the mouse MOE 1 Mb with the EPO track. For each of the two BLAST outputs, the 100 best-matching subjects were stored as FASTA files. On these, we ran a first FIMO screen using every PSWM (α = 0.05); only those FASTA entries possessing significant hits for specific matrices that do not contain sequence information from [28, 42] were retained for further analyses. On these, a second round of FIMO investigations was performed (for all PSWMs, with an α value of 0.05). Such second-round predicted elements were then BLAST-searched on known elements, namely H, P, J and all elements proposed in [28]. This let us understand whether enhancers found by [28] or [42] were rediscovered by the procedure.

### Data plotting

We used local scripting to draft line charts representing the effect of threshold changes on the number of clusters/solitary genes, and to obtain bar charts reporting number of genes per cluster and cluster gene density; line charts with Bayesian information criterion values for Ckmeans.1d.dp were produced with the R function plotBIC. Chromosome maps were outlined on Idiographica [85]; when needed, mapping coordinates of known mouse enhancers were updated to GRCm38 via UCSC LiftOver [86]. Details on single *loci* or candidate elements were prepared with MacVector. Graphical renderings of PSWM were directly outputted by MEME.

## Additional files

Additional file 1: **Figure S1.** K-means clustering produces an alternative architecture for the mouse main olfactory epithelium (MOE) list. **A.** Single-chromosome line charts reporting (in dark cyan) the ratio between the value of the Bayesian information criterion (BIC) and the number of genes (n), for each imposed number of *loci* (k). k* indicates (in black) the ideal k value for k-means clustering; k^~^ indicates (in red) the number of *loci* found, for the same chromosome, by our distance-based clustering method (for threshold = 1 Mb). Small boxes (indicated by black arrows) magnify graph areas around k* and k^~^. **B.** Chromosome charts for the mouse MOE list, using a 1 Mb cutoff (left) or k-means clustering (right). Distance-based *loci* are reported as magenta intervals (for clusters) or green squares (for solitary genes); solitary genes are annotated on their sense strand (be it plus, +, or minus, -). k-means-based *loci* are invariably reported as dark cyan intervals. A location containing oversplit clusters is magnified (black shadowed box). Chromosome bands represent Giemsa staining. (TIFF 7829 kb)

Additional file 2: **Figure S2.** Graphical representations of position-specific weight matrices (PSWMs) used to predict elements. Matrices 1 to 3 are derived from mouse and rat core element sequences discovered prior to 2014 (i.e. “early”); PSWMs 4 and 5 were obtained from all known mouse elements for class II OR genes (that is, “late”), while 6 and 7 reproduce class J-like (J) enhancers as found in different Muridae *taxa*. Remaining matrices represent single transcription factor binding sites, either for homeodomain (HD, PSWM 8) or olfactory/early B factors (O/E, matrices 9 to 12). (TIFF 7831 kb)

Additional file 3: Detailed information about *locus* composition at various threshold distances. Text files (.txt) detailing on *locus* composition, at a given threshold distance (in Mb), for different gene lists. File names are composed by list type (either: main olfactory epithelium, MOE; olfactome; vomeronasal organ, VNO; T-cell receptor genes, “t_cell_receptor”), species and threshold value (periods in numerical values being replaced by underscores, “_”). A “NO-PSEUDO” at the beginning of the file name indicates that the gene list originating the file is devoid of pseudogene-flagged entries. Clusters are presented from the richest (in terms of number of genes) to miniclusters; for each *locus*, the average intergenic distance (in Kb) is reported. Solitary genes are listed after clusters. Text files are provided as a single compressed (.zip) file available in the Open Science Framework repository, see paragraph “Availability of data and material”. (ZIP 719 kb)

Additional file 4: Annotation tracks highlighting clusters and solitary genes for selected genomic architectures. BED-formatted text files (.bed) annotating *loci*, at a given threshold distance (in Mb), for different gene lists. File names are composed by list type (either: main olfactory epithelium, MOE; olfactome; vomeronasal organ, VNO; T-cell receptor genes, “t_cell_receptor”), species and threshold value (periods in numerical values being replaced by underscores, “_”). A “NO-PSEUDO” at the beginning of the file name indicates that the gene list originating the file is devoid of pseudogene-flagged entries. Text files are provided as a single compressed (.zip) file available in the Open Science Framework repository, see paragraph “Availability of data and material”. (ZIP 155 kb)

Additional file 5: Annotation tracks containing evolutionarily conserved stretches among *loci* of selected genomic architectures. BED-formatted text files (.bed) annotating, at threshold distance = 1 Mb, broadened conserved stretches within (or nearby) *loci* of some notable genomic architectures. File names are composed by list type (either: main olfactory epithelium, MOE; olfactome; vomeronasal organ, VNO; T-cell receptor genes, “t_cell_receptor”), species and threshold value (invariably “1_0Mb”). A “NO-PSEUDO” in the file name indicates that the gene list originating the file is devoid of pseudogene-flagged entries. A “CONSERVED” at the beginning of each file name differentiates these BED files from those found in Additional files 4 and 7. Text files are provided as a single compressed (.zip) file available in the Open Science Framework repository, see paragraph “Availability of data and material”. (ZIP 2843 kb)

Additional file 6: Position-specific weight matrices (PSWMs) used to predict elements. Matrices are presented as MEME-formatted text files (.txt). Each file name reports the numeric identifier of the corresponding PSWM. Text files are provided as a single compressed (.zip) file available in the Open Science Framework repository, see paragraph “Availability of data and material”. (ZIP 37 kb)

Additional file 7: Annotation tracks listing all predicted enhancers for genomic architecture in which an element search was performed. BED-formatted text files (.bed) annotating, at threshold distance = 1 Mb, putative elements found within (or nearby) *loci* of selected genomic architectures. File names are composed by list type (either: main olfactory epithelium, MOE; olfactome; vomeronasal organ, VNO; T-cell receptor genes, “t_cell_receptor”), species and threshold value (invariably “1_0Mb”); “BLAST” marks the MOE putative enhancer list obtained through the BLAST-derived pipeline variant. A “NO-PSEUDO” in the file name indicates that the gene list originating the file is devoid of pseudogene-flagged entries. A “CAND-EL” at the beginning of each file name differentiates these BED files from those found in Additional files 4 and 5. Text files are provided as a single compressed (.zip) file available in the Open Science Framework repository, see paragraph “Availability of data and material”. (ZIP 7 kb)

Additional file 8: **Table S1.** *Locus* composition for some of the genomic architectures evaluated. List types are main olfactory epithelium (MOE), vomeronasal organ (VNO), olfactome or T-cell receptor (TCR) genes; a “NO-PSEUDO” preceding the name of a list indicates that such list is devoid of pseudogene-flagged entries. Gene families are odorant receptor (OR) genes, vomeronasal receptor (VR) genes, trace amine-associated receptor (TAAR) genes, formyl peptide receptor (FPR) genes and TCR genes. For each list, the total number of *loci* can be easily obtained by summing up cluster number and solitary gene number (i.e. values reported outside of parentheses). Cluster number comprises that of miniclusters; genomic coordinates and genes composing miniclusters are noted down explicitly, respectively in round and square brackets. Mixed clusters should be intended as those locations made up by members belonging to more than one of the above-mentioned gene families. (PDF 105 kb)

## Data Availability

Additional files 3, 4, 5, 6 and 7 are available in the Open Science Framework repository, doi:10.17605/OSF.IO/7MRHU (direct link https://osf.io/7mrhu/). All other relevant data generated for the study are included within the manuscript and its supplementary information.

## Abbreviations

FPR: Formyl peptide receptor
GPCR: G protein-coupled receptor
HD: Homeodomain
MOE: Main olfactory epithelium
O/E: Olfactory/early B
OR: Odorant receptor
OSN: Olfactory sensory neuron
PSWM: Position-specific weight matrix
TAAR: Trace amine-associated receptor
TCR: T-cell receptor
TFBS: Transcription factor binding site
VNO: Vomeronasal organ
VR: Vomeronasal receptor
VSN: Vomeronasal sensory neuron
