# Supplementary figures and images for: A purely bioinformatic pipeline for the prediction of mammalian odorant receptor gene enhancers
Source: BMC Bioinformatics. 2019 Sep 14;20:474. doi: 10.1186/s12859-019-3012-1 (PMC6744719; doi:10.1186/s12859-019-3012-1)

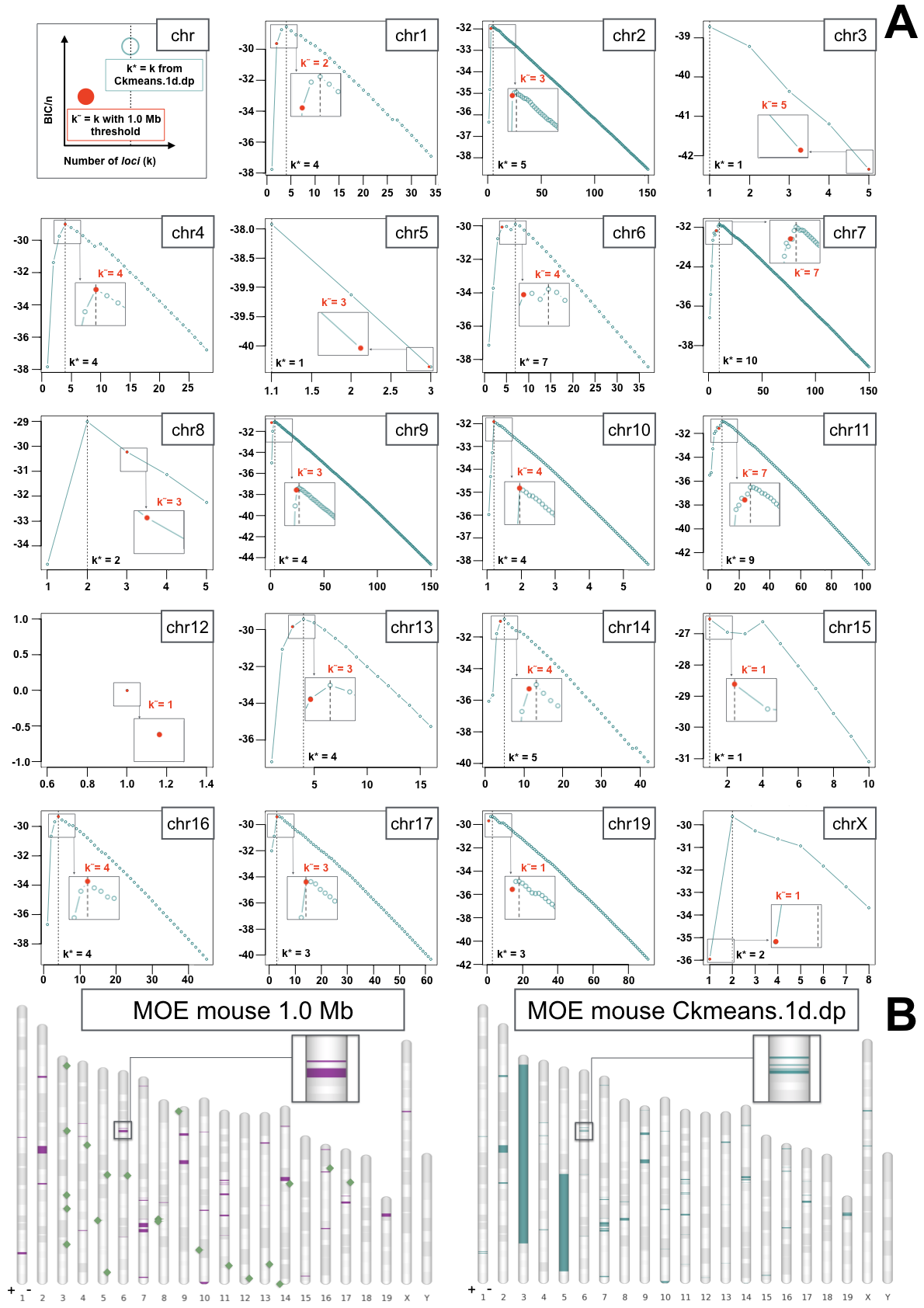

Supplement: Supplementary file 1 — Figure S1. K-means clustering produces an alternative architecture for the mouse main olfactory epithelium (MOE) list. A. Single-chromosome line charts reporting (in dark cyan) the ratio between the value of the Bayesian information criterion (BIC) and the number of genes (n), for each imposed number of loci (k). k* indicates (in black) the ideal k value for k-means clustering; k~ indicates (in red) the number of loci found, for the same chromosome, by our distance-based clustering method (for threshold = 1 Mb). Small boxes (indicated by black arrows) magnify graph areas around k* and k~. B. Chromosome charts for the mouse MOE list, using a 1 Mb cutoff (left) or k-means clustering (right). Distance-based loci are reported as magenta intervals (for clusters) or green squares (for solitary genes); solitary genes are annotated on their sense strand (be it plus, +, or minus, -). k-means-based loci are invariably reported as dark cyan intervals. A location containing oversplit clusters is magnified (black shadowed box). Chromosome bands represent Giemsa staining. (TIFF 7829 kb) [file 12859_2019_3012_MOESM1_ESM.tiff]

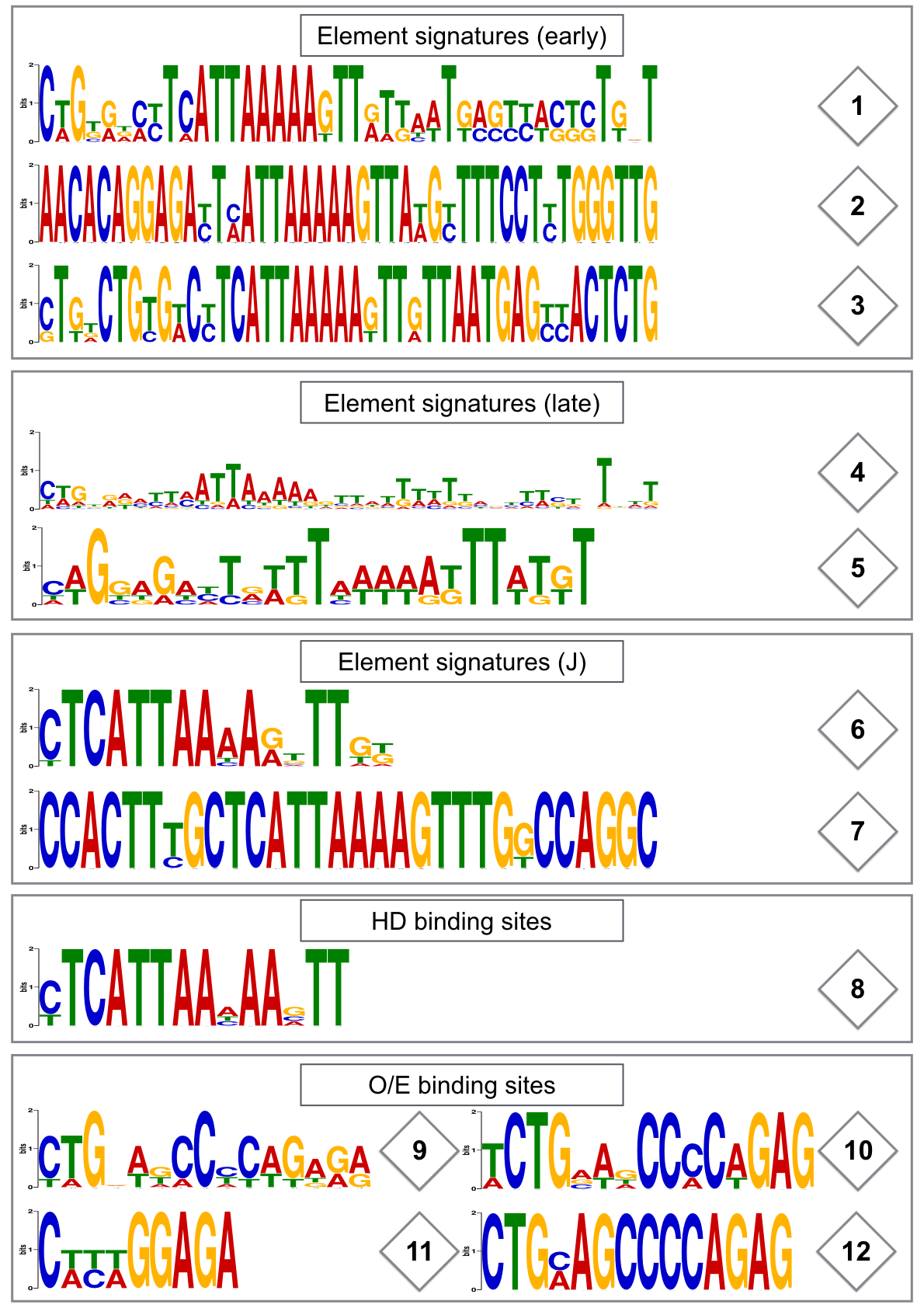

Supplement: Supplementary file 2 — Figure S2. Graphical representations of position-specific weight matrices (PSWMs) used to predict elements. Matrices 1 to 3 are derived from mouse and rat core element sequences discovered prior to 2014 (i.e. “early”); PSWMs 4 and 5 were obtained from all known mouse elements for class II OR genes (that is, “late”), while 6 and 7 reproduce class J-like (J) enhancers as found in different Muridae taxa. Remaining matrices represent single transcription factor binding sites, either for homeodomain (HD, PSWM 8) or olfactory/early B factors (O/E, matrices 9 to 12). (TIFF 7831 kb) [file 12859_2019_3012_MOESM2_ESM.tiff]
