# Supplementary material for: A purely bioinformatic pipeline for the prediction of mammalian odorant receptor gene enhancers
Source: BMC Bioinformatics. 2019 Sep 14;20:474. doi: 10.1186/s12859-019-3012-1 (PMC6744719; doi:10.1186/s12859-019-3012-1)
Supplement: Supplementary file 8 — Table S1. Locus composition for some of the genomic architectures evaluated. List types are main olfactory epithelium (MOE), vomeronasal organ (VNO), olfactome or T-cell receptor (TCR) genes; a “NO-PSEUDO” preceding the name of a list indicates that such list is devoid of pseudogene-flagged entries. Gene families are odorant receptor (OR) genes, vomeronasal receptor (VR) genes, trace amine-associated receptor (TAAR) genes, formyl peptide receptor (FPR) genes and TCR genes. For each list, the total number of loci can be easily obtained by summing up cluster number and solitary gene number (i.e. values reported outside of parentheses). Cluster number comprises that of miniclusters; genomic coordinates and genes composing miniclusters are noted down explicitly, respectively in round and square brackets. Mixed clusters should be intended as those locations made up by members belonging to more than one of the above-mentioned gene families. (PDF 105 kb) [file 12859_2019_3012_MOESM8_ESM.pdf]

Supplementary Table S1

List	Cluster number (of which mixed)	Minicluster number	Solitary gene number (of which pseudogenes)	Minicluster identity and genes	Solitary gene identity
MOE mouse 0.1 Mb threshold	61 (0)	9	31 (14)	cluster12 (chr6:40542171-40574343), [*Olfr461*, *Olfr460*]; cluster18 (chr7:84853553-84920861), [*Olfr291*, *Olfr290*]; cluster20 (chr7:99730273-99769321), [*Olfr520*, *Olfr521*]; cluster33 (chr10:78517428-78618074), [*Olfr1358*, *Olfr1357*]; cluster46 (chr13:65138089-65156152), [*Olfr465-ps1*, *Olfr466*]; cluster47 (chr14:14322828-14410095), [*Olfr31*, *Olfr721-ps1*]; cluster58 (chr19:12670439-12683851), [*Olfr1442*, *Olfr1443*]; cluster60 (chrX:49672333-49735059), [*Olfr1320*, *Olfr1321*]; cluster61 (chrX:74569153-	*Olfr15*, *Olfr16*, *Olfr19*, *Olfr49*, *Olfr210-ps1*, *Olfr221*, *Olfr241-ps1*, *Olfr266*, *Olfr267*, *Olfr277-ps1*, *Olfr289-ps1*, *Olfr369-ps1*, *Olfr370*, *Olfr371*, *Olfr375-ps1*, *Olfr459*, *Olfr717-ps1*, *Olfr718-ps1*, *Olfr719-ps*, *Olfr720*, *Olfr750*, *Olfr752-ps1*, *Olfr1322*, *Olfr1323*, *Olfr1324*, *Olfr1367*, *Olfr1397-ps1*, *Olfr1398-ps1*, *Olfr1401-ps1*, *Olfr1402*, *Olfr1556-ps1*

				74597578), [*Olfr1326-ps1*, *Olfr1325*]	
MOE mouse 0.2 Mb threshold	50 (0)	5	26 (14)	cluster10 (chr6:40542171-40574343), [*Olfr461*, *Olfr460*]; cluster14 (chr7:84853553-84920861), [*Olfr291*, *Olfr290*]; cluster16 (chr7:99730273-99769321), [*Olfr520*, *Olfr521*]; cluster26 (chr10:78517428-78618074), [*Olfr1358*, *Olfr1357*]; cluster38 (chr13:65138089-65156152), [*Olfr465-ps1*, *Olfr466*]	*Olfr15*, *Olfr19*, *Olfr49*, *Olfr210-ps1*, *Olfr221*, *Olfr241-ps1*, *Olfr266*, *Olfr267*, *Olfr277-ps1*, *Olfr289-ps1*, *Olfr369-ps1*, *Olfr370*, *Olfr371*, *Olfr375-ps1*, *Olfr459*, *Olfr717-ps1*, *Olfr718-ps1*, *Olfr719-ps*, *Olfr750*, *Olfr752-ps1*, *Olfr1324*, *Olfr1397-ps1*, *Olfr1398-ps1*, *Olfr1401-ps1*, *Olfr1402*, *Olfr1556-ps1*
MOE mouse 0.5 Mb threshold	46 (0)	4	22 (14)	cluster10 (chr6:40542171-40574343), [*Olfr461*, *Olfr460*]; cluster14 (chr7:84853553-84920861), [*Olfr291*, *Olfr290*]; cluster16 (chr7:99730273-99769321), [*Olfr520*, *Olfr521*]; cluster35 (chr13:65138089-65156152), [*Olfr465-ps1*, *Olfr466*]	*Olfr19*, *Olfr49*, *Olfr210-ps1*, *Olfr241-ps1*, *Olfr266*, *Olfr267*, *Olfr277-ps1*, *Olfr289-ps1*, *Olfr369-ps1*, *Olfr370*, *Olfr371*, *Olfr375-ps1*, *Olfr459*, *Olfr717-ps1*, *Olfr718-ps1*, *Olfr719-ps*, *Olfr752-ps1*, *Olfr1397-ps1*, *Olfr1398-ps1*, *Olfr1401-ps1*, *Olfr1402*, *Olfr1556-ps1*
MOE mouse 1 Mb threshold	42 (0)	4	21 (14)	cluster9 (chr6:40542171-40574343), [*Olfr461*, *Olfr460*]; cluster13 (chr7:84853553-84920861), [*Olfr291*, *Olfr290*]; cluster15 (chr7:99730273-	*Olfr19*, *Olfr49*, *Olfr210-ps1*, *Olfr241-ps1*, *Olfr266*, *Olfr267*, *Olfr277-ps1*, *Olfr289-ps1*, *Olfr369-ps1*, *Olfr370*, *Olfr371*, *Olfr375-ps1*, *Olfr717-ps1*, *Olfr718-ps1*, *Olfr719-ps*,

				99769321), [*Olfr520*, *Olfr521*]; cluster32 (chr13:65138089-65156152), [*Olfr465-ps1*, *Olfr466*]	*Olfr752-ps1*, *Olfr1397-ps1*, *Olfr1398-ps1*, *Olfr1401-ps1*, *Olfr1402*, *Olfr1556-ps1*
NO-PSEUDO MOE mouse 1 Mb threshold	41 (0)	6	8 (0 by definition)	cluster9 (chr6:40542171-40574343), [*Olfr461*, *Olfr460*]; cluster13 (chr7:84853553-84920861), [*Olfr291*, *Olfr290*]; cluster15 (chr7:99730273-99769321), [*Olfr520*, *Olfr521*]; cluster27 (chr11:52144593-52213987), [*Olfr1373*, *Olfr1371*]; cluster32 (chr14:14171738-14331791), [*Olfr720*, *Olfr31*]; cluster35 (chr16:3591042-3844747), [*Olfr161*, *Olfr15*]	*Olfr19*, *Olfr49*, *Olfr266*, *Olfr267*, *Olfr370*, *Olfr371*, *Olfr466*, *Olfr1402*
Olfactome mouse 1 Mb threshold	59 (9, of which 1 FPR/VR-mixed and 8 OR/VR-mixed)	4	35 (23)	cluster12 (chr4:156331103-156354578), [*Vmn2r-ps159*, *Vmn2r125*]; cluster16 (chr6:40542171-40574343), [*Olfr461*, *Olfr460*]; cluster28 (chr7:99730273-99769321), [*Olfr520*, *Olfr521*]; cluster45 (chr13:65138089-65156152), [*Olfr465-ps1*, *Olfr466*]	*Olfr19*, *Olfr49*, *Olfr210-ps1*, *Olfr241-ps1*, *Olfr266*, *Olfr267*, *Olfr277-ps1*, *Olfr289-ps1*, *Olfr369-ps1*, *Olfr370*, *Olfr371*, *Olfr375-ps1*, *Olfr717-ps1*, *Olfr718-ps1*, *Olfr719-ps*, *Olfr1397-ps1*, *Olfr1398-ps1*, *Olfr1401-ps1*, *Olfr1402*, *Olfr1556-ps1*, *V1rg10*, *Vmn1r90*, *Vmn1r239-ps*, *Vmn1r-ps1*, *Vmn2r118*, *Vmn2r120*, *Vmn2r121*, *Vmn2r-ps2*, *Vmn2r-*

					ps24, *Vmn2r-ps61*, *Vmn2r-ps88*, *Vmn2r-ps89*, *Vmn2r-ps94*, *Vmn2r-ps104*, *Vmn2r-ps139*
Olfactome rat 1 Mb threshold	72 (9, of which 2 FPR/VR-mixed, 1 FPR/OR/VR-mixed and 6 OR/VR-mixed)	17	19 (1)	cluster1 (chr1:744616-813517), [*Vom2r6*, *Vom2r5*]; cluster7 (chr1:116152667-116885644), [*Fpr3*, *Vom2r25*]; cluster9 (chr1:164705604-164756039), [*Olr36*, *Olr37*]; cluster18 (chr2:168228099-168260536), [*Vom1r56*, *Vom1r55*]; cluster25 (chr4:13564879-13645351), [*Olr987*, *Olr990*]; cluster28 (chr4:119398198-119466491), [*Vom1r101*, *Vom1r102*]; cluster33 (chr5:64614755-64622360), [LOC500460, *Olr841*]; cluster43 (chr9:7643533-7815144), [*Vom2r76*, *Vom2r77*]; cluster45 (chr9:101222846-101269843), [*Vom1r64*, *Olr178*]; cluster52 (chr10:75186572-75202030), [*Olr1521*, *Olr1522*]; cluster54 (chr11:23057512-23063414), [LOC103693533, LOC103690036]; cluster57	LOC100909435, LOC100912028, LOC103693627, *Olr390*, *Olr392*, *Olr799*, *Olr854*, *Olr1646*, *Olr1667*, *Vom1r26*, *Vom1r50*, *Vom1r70*, *Vom1r89*, *Vom2r9*, *Vom2r38*, *Vom2r49*, *Vom2r57*, *Vom2r75*, *Vom2r79*

				(chr11:55474820-55498163), [*Olr1453*, LOC108348268]; cluster60 (chr12:19753207-21077539), [*Vom2r62*, *Vom2r64*]; cluster61 (chr13:9464906-9480933), [LOC100910928, LOC100910887]; cluster67 (chr17:2690062-2705123), [*Olr1653*, *Olr1652*]; cluster69 (chr19:27534854-28378118), [*Olr1666*, *Vom1r21*]; cluster71 (chrX:95344825-95353344), [*Olr10*, *Olr11*]	
Olfactome human 1 Mb threshold	65 (7, of which 1 OR/TAAR-mixed and 6 OR/VR-mixed)	15	38 (34)	cluster2 (chr1:147419050-147446133), [*OR13Z1P*, *OR13Z2P*]; cluster8 (chr3:13926813-14089871), [*VN1R20P*, *VN1R21P*]; cluster9 (chr3:75356516-75599673), [*OR7E22P*, *OR7E121P*]; cluster16 (chr7:56559221-57423725), [*VN1R25P*, *VN1R28P*]; cluster18 (chr7:97946987-97967074), [*OR7E7P*, *OR7E38P*]; cluster24 (chr9:38504447-39039594), [*VN1R48P*, *VN2R3P*]; cluster29 (chr10:45258185-45315608), [*OR6D1P*,	*OR1X1P*, *OR1X5P*, *OR2AE1*, *OR2AL1P*, *OR2BH1P*, *OR2K2*, *OR4F1P*, *OR4F2P*, *OR4F7P*, *OR4F21*, *OR5M14P*, *OR6R2P*, *OR7A19P*, *OR7E14P*, *OR7E23P*, *OR7E94P*, *OR7E100P*, *OR7E101P*, *OR7E102P*, *OR7E122P*, *OR7E129P*, *OR7E136P*, *OR7H2P*, *OR7K1P*, *OR8R1P*, *OR11H1*, *OR11I1P*, *VN1R7P*, *VN1R9P*, *VN1R46P*, *VN1R51P*, *VN1R53P*, *VN1R54P*, *VN1R96P*, *VN1R108P*, *VN1R110P*, *VN2R1P*, *VN2R10P*

				OR13A1]; cluster35 (chr11:67735608-67975243), [*OR7E11P*, *OR7E1P*]; cluster38 (chr11:86832540-86857947), [*OR7E13P*, *OR7E2P*]; cluster41 (chr12:52079696-52092285), [*OR7E47P*, *OR7E47P*]; cluster44 (chr13:63742341-63837456), [*OR7E156P*, *OR7E104P*]; cluster45 (chr13:67902208-67911951), [*OR7E111P*, *OR7E33P*]; cluster48 (chr15:20960675-20970215), [*OR11J2P*, *OR11J5P*]; cluster54 (chr17:58155133-58170579), [*OR4D1*, *OR4D2*]; cluster62 (chr19:54320204-54648853), [*VN1R104P*, *VN1R105P*]	
